# Prognostic value of pre-treatment FDG PET/CT SUVmax for metastatic lesions in de novo metastatic nasopharyngeal carcinoma following chemotherapy and locoregional radiotherapy

**DOI:** 10.1186/s40644-023-00536-z

**Published:** 2023-02-24

**Authors:** Wenbin Yan, Chunhua Sun, Xiaomin Ou, Chaosu Hu

**Affiliations:** 1 Department of Radiation Oncology, Fudan University Shanghai Cancer Center, 270 Dong An Road, Shanghai, 200032 China; 2 Department of Oncology, Shanghai Medical College, Fudan University, Shanghai, 200032 China; 3 Shanghai Clinical Research Center for Radiation Oncology, Shanghai, 200032 China; 4 Shanghai Key Laboratory of Radiation Oncology, Shanghai, 200032 China; 5 Department of Radiation Oncology, The Affiliated Wuxi Second People’s Hospital of Nanjing Medical University, Wuxi, China

**Keywords:** PET/CT, SUVmax, Metastatic, Nasopharyngeal carcinoma

## Abstract

**Background:**

To explore the prognostic role of FDG PET/CT maximal standard uptake values of metastatic lesions (SUVmax-M) in patients with de novo metastatic nasopharyngeal carcinoma (mNPC) following palliative chemotherapy and locoregional radiotherapy (LRRT).

**Methods:**

We retrospectively collected the information of 86 eligible patients between Jan 2012 and Oct 2020. All the parameters involving SUVmax and serum lactate dehydrogenase (LDH) at diagnosis were evaluated and cutoff values were determined by the maximum log-rank statistic method. The multivariate analysis was performed using Cox proportional hazards regression to identify the independent prognostic factors associated with overall survival (OS). All estimated survival rates were conducted with Kaplan–Meier method.

**Results:**

Median survival and progression time in the cohort were 38.2 and 13.9 months, respectively. The univariable analysis showed that male, number of metastatic sites ≥ 4, presence of liver, serum LDH ≥ 229, SUVmax-M ≥ 10, SUVmax-M-sum ≥ 10, and SUVmax-M-mean ≥ 8.8 were significant prognostic factors. Five variables were identified after LASSO regression and entered into the multivariate analysis. Furthermore, liver involvement (*P* = 0.039), elevated LDH (≥ 229) (*P* = 0.05) and higher SUVmax-M (≥ 10) (*P* = 0.004) were significantly associated with worse OS.

**Conclusion:**

The high SUVmax of metastatic lesions (≥ 10), liver involvement, and elevated serum LDH (≥ 229) at diagnosis could independently predict poor survival for de novo mNPC patients treated with palliative chemotherapy following LRRT.

## Introduction

Nasopharyngeal carcinoma (NPC) is a prevalent malignancy arising from the nasopharyngeal mucosa in southeast Asia [1]. Histologically three subtypes were classified according to the World Health Organization, namely keratinizing, and non-keratinizing (differentiated and undifferentiated). The non-keratinizing carcinoma comprised the major histology in endemic area, which was closely associated with Epstein-Barr virus (EBV) infection [2]. Unlike squamous cell carcinoma of the head and neck, NPC was characterized with high sensitivity to radiation and chemotherapy. Excellent local control and survival outcome could be achieved following intensive chemoradiotherapy (CRT) with the 5-year distant failure around 20% [3, 4]. Notably, nearly 8% [5] of the NPC patients presented with synchronous distant metastasis at diagnosis.

Multiple doublets chemotherapy was recommended as the first-line therapeutic approach for de novo metastatic NPC (mNPC). A prospective randomized trial [6] has demonstrated better overall survival (OS) and treatment response of gemcitabine and cisplatin (GP) compared with fluorouracil and cisplatin (PF) regimen in metastatic NPC. Additionally, the reported overall response (complete and partial response) could approach 90% for the chemo-naïve mNPC patients [7]. Thus, combining locoregional radiotherapy (LRRT) with chemotherapy offered superior survival benefit than palliative chemotherapy alone in the responsive cases [8]. However, the prognosis of de novo mNPC can be extremely diversified due to the various clinical features. Therefore, cooperating with relevant factors for accurately predicting the survival outcome was helpful for personalizing treatment strategy in de novo mNPC.

^18^F-fluorodeoxyglucose positron emission tomography (FDG PET/CT) has illustrated great efficacy in detecting distant metastasis of NPC at diagnosis [9, 10]. The higher pretreatment maximum of standardized uptake value (SUVmax) in primary tumor and lymph node was correlated with poor outcome in non-disseminated NPC [11–13]. Additionally, the SUVmax before and after radiotherapy appeared to be predictive factors for poor prognosis [14] in locally advanced NPC. The SUVmax of metastatic lesions (SUVmax-M) was also correlated with worse OS in some other metastatic tumors [15, 16]. The synchronous metastatic lesions along with the primary tumor and cervical nodes emerged with different metabolic features in the patients with de novo mNPC. However, the predictive value of SUVmax-M in de novo mNPC patients following palliative chemotherapy and LRRT was not conclusive.

In this study, we aimed to evaluate the metabolic activity measured by pretreatment FDG PET/CT in the de novo mNPC, and explore the potential prognostic role of the SUVmax-M in the patients treated with chemotherapy following LRRT.

## Materials and methods

### Patients selection

Clinical data of patients pathologically confirmed as NPC treated at Fudan University Shanghai Cancer Center (FUSCC) between Jan 2012 and Oct 2020 were retrospectively collected. Inclusion criteria included: 1) patients with de novo metastatic nasopharyngeal carcinoma (mNPC) following palliative chemotherapy and locoregional radiotherapy (LRRT); 2) underwent ^18^F-FDG PET/CT at diagnosis;. Those patients without PET, received only palliative chemotherapy, or with follow-up information missing were excluded from the study.

### ^18^F-FDG PET/CT

The procedure of the FDG PET/CT performed following the recommendation of the European Association of Nuclear Medicine (EANM) [17].All the patients underwent whole-body FDG PET/CT scanning by Siemens biograph 16HR PET/CT scanner (Knoxville, Tennessee, USA) in our center. Before PET/CT, all patients were requested to fast for at least 4 h. The serum glucose levels were measured after the tracer injection, and the schedule was conducted when the level was under 10 mmol/L. Imaging scanning was initiated 1 h after the administration of tracer, and the scans were acquired from the base of the skull to the upper thighs within 2–3 min per bed position. CT scanning was first performed (with 120 kV, 80 ~ 250 mA, pitch 3.6, rotation time 0.5), and a PET emission scan that covered the identical transverse field of view was obtained. PET image data sets were reconstructed iteratively by applying CT data for attenuation correction, and coregistered images were confirmed on a workstation. The maximum of standard uptake value (SUVmax) for primary tumor, cervical lymph nodes and metastatic lesions were evaluated by individual region of interest (ROI) around the lesions. SUVmax metastasis (SUVmax-M) was defined as the value of the hottest metastatic lesion. SUVmax-M-sum was calculated as the sum of the SUVmax for each metastatic site. SUVmax-M-mean was defined as SUVmax-M-sum/number of metastatic sites.

### Treatment approach

All the patients received initial chemotherapy after the diagnosis of de novo mNPC. The regimens including gemcitabine and cisplatin (GP), docetaxel and cisplatin (TP), or cisplatin and 5-fluorouracil (PF), or docetaxel, cisplatin and 5-fluorouracil (TPF) were administrated for 3–10 cycles. Clinical treatment response was evaluated every two cycles using CT or MRI examination according to the Response Evaluation Criteria in Solid Tumors (RECIST 1.1). LRRT was delivered using intensity-modulated radiation therapy (IMRT) or three-dimensional conformal radiation therapy (3D-CRT) once daily for five times per week. The targets volumes were delineated based on the pre-chemotherapy imaging data. The prescribed doses were 60–70.4 Gy for the planning target volume (PTV), 54-60 Gy for the PTV1, and 51-54 Gy for the PTV2 of the involved lymph nodal area in 28 to 35 fractions.

### Follow-up and outcome

The patients were followed up regularly every 3 months for the first 3 years after treatment, and every 6 months after 3 years. The usual evaluation included physical examination, head and neck MRI, chest and abdominal CT, and whole-body bone emission computed tomography scanning or PET-CT scans. The primary endpoint for this study was overall survival (OS) which was calculated from the date of initial diagnosis to the date of death from any cause, or last follow-up. Progression-free survival (PFS) was defined as the period between date of initial diagnosis and the time of disease progression or death from any cause.

### Statistical analysis

The clinical characteristics of patients were summarized with the categorial variables displayed as amounts and proportions (%), while continuous variables as median and range. The continuous variables were converted into binary variables with the optimal cutoff values determined by maximally selected rank statistics method [18]. Univariable analysis for associations between the selecting variables and OS were analyzed with the log-rank method. And multivariate analysis (MVA) was conducted using the Cox proportional hazards model to identify independent prognostic factors. Survival outcome was evaluated with Kaplan–Meier survival curves.

All statistical analyses were performed by R software version 4.1.5 (Vienna, Austria), and two-tailed *P* < 0.05 was considered statistically significant.

## Results

### Patients and treatment characteristics

**Fig. 1:**
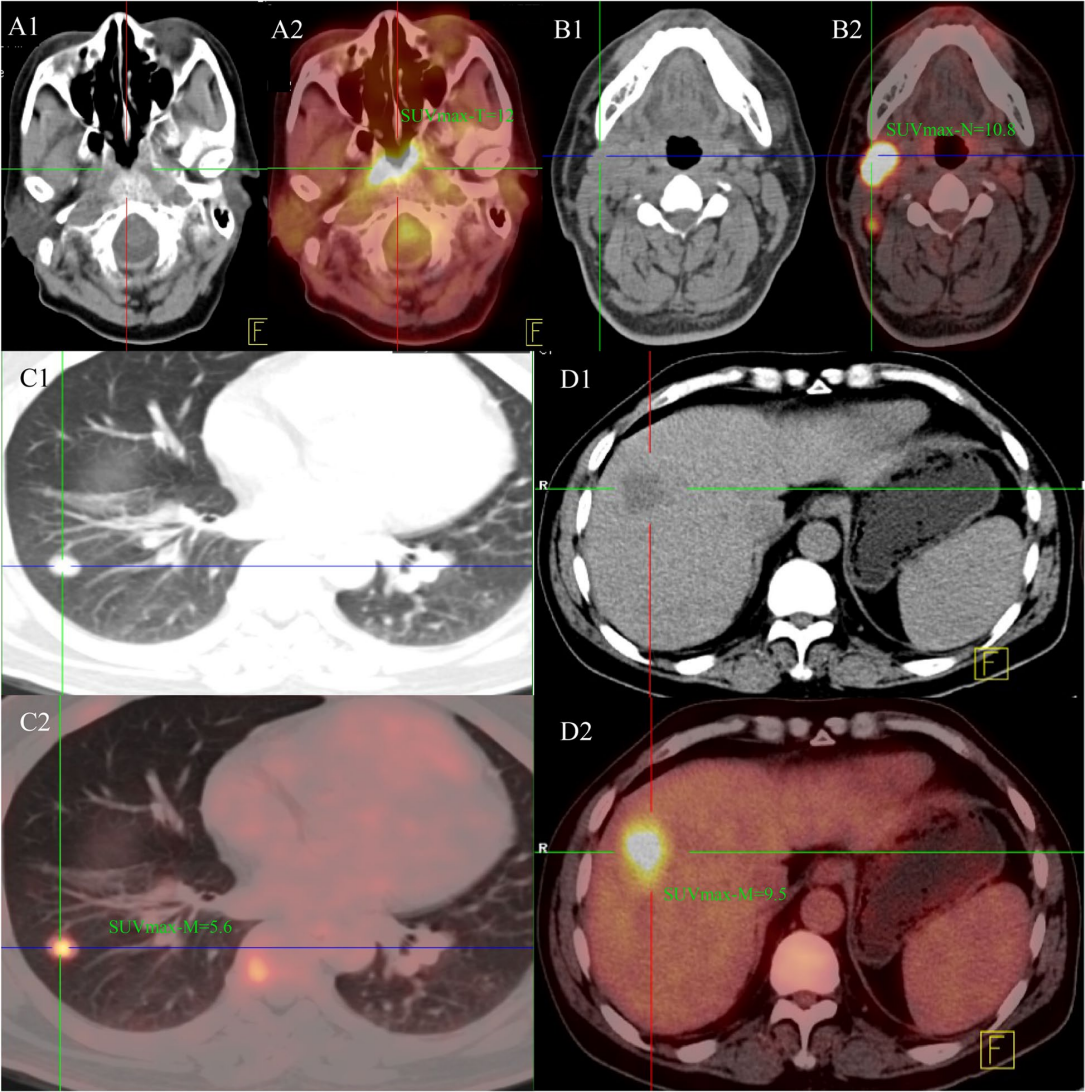
A 42 years old male with primary tumor of nasopharyngeal, right lymph node involvement, along with distant lung and liver metastasis. A1-2) SUVmax of primary tumor was 12. B1-2) SUVmax-N was 10.8, C1-2) distant metastasis of lung, SUVmax-M = 5.6, D1-2) distant liver metastasis, SUVmax = 9.5, SUVmax-M-sum = 15.1, SUVmax-M-mean = 7.6

A total of 86 patients met the inclusion in this cohort study. The median age at diagnosis was 45 years, and 76 (88.4%) patients were males. 15.1% (*n* = 13) of the patients were pathologically differentiated non-keratinizing (WHO II type) whereas 84.9% of cases were undifferentiated non-keratinizing (WHO III type). Most of the involved distant metastasis was bone (*n* = 61, 70.9%) followed liver metastasis (*n* = 21, 24.4%). 74.4% of the eligible patients presented with single distant organ metastasis at diagnosis. The treatment strategy for the patients included palliative chemotherapy (PCT) followed by radiotherapy (*n* = 70, 81.4%) and PCT plus concurrent chemoradiotherapy (CCRT) (*n* = 16, 18.6%). Figure 1 showed the example of patients with primary tumor, neck involvement and distant metastasis at diagnosis. More details about the clinical characteristics are summarized in Table 1.

**Table 1:** Details of the characteristics of the eligible patients (*n* = 86)

	*N* = 86 (%)
Gender	
Male	76 (88.4)
Female	10 (11.6)
Age	45 (20–70)
Pathology	
WHO II	13 (15.1)
WHO III	73 (84.9)
T stage	
T1	8 (9.3)
T2	29 (33.7)
T3	30 (34.9)
T4	19 (22.1)
N stage	
N1	10 (11.6)
N2	39 (45.3)
N3	37 (43.1)
Metastatic organ	
Bone	61 (70.9)
Liver	21 (24.4)
Lung	8 (9.3)
Node	23 (26.7)
Metastatic sites	
Single	64 (74.4)
Multiple	22 (25.6)
EBV-DNA (copies/ml)	
< 500	5 (5.8)
> 500	25 (29.1)
Missing	56 (65.1)
Lactate dehydrogenase (U/L)	
< 250	55 (63.9)
≥ 250	31 (36.1)
PET/CT SUVmax	
Primary tumor	11.4 (3.7–35.7)
Lymph node	11.3 (2.3–36.1)
Metastatic site	9.25 (2–20.6)
Bone	8.8 (2–20.6)
Liver	7.9 (4.4–20.5)
Lung	7.9 (2–10.6)
Node	9.25 (2–20.6)
Treatment	
PCT + RT	70 (81.4)
PCT + CCRT	16 (18.6)
Response to CT (primary tumor)	
Complete response (CR)	2 (2.3)
Partial response (PR)	75 (87.2)
Stable disease (SD)	6 (7.0)
Progression disease (PD)	3 (3.5)
Response to CT (metastatic site)	
CR	3 (3.5)
PR	23 (90.5)
SD	10 (90.5)
PD	4 (9.54)
Not reported	46 (9.54)

*Abbreviations: PCT* palliative chemotherapy, *RT* radiotherapy, *CCRT* concurrent chemoradiotherapy

### Follow-up and outcomes

After a median follow-up time of 82.1 months (95% CI: 73-107months), the estimated 2, and 5-year OS was 70.6% (95%CI 61.5%-81%), and 37.1% (95% CI 27.8%-49.6%), respectively. The median survival time was 38.2 months (95%CI 30.4-57.3 months). The 2-year PFS of the study cohort was 36.3% (95%CI 27.3%-48.3%) with the median progression time as 13.9 months (95% CI 11.1-22.7 months).

### Definitions for the parameters of the FDG PET/CT

**Fig. 2:**
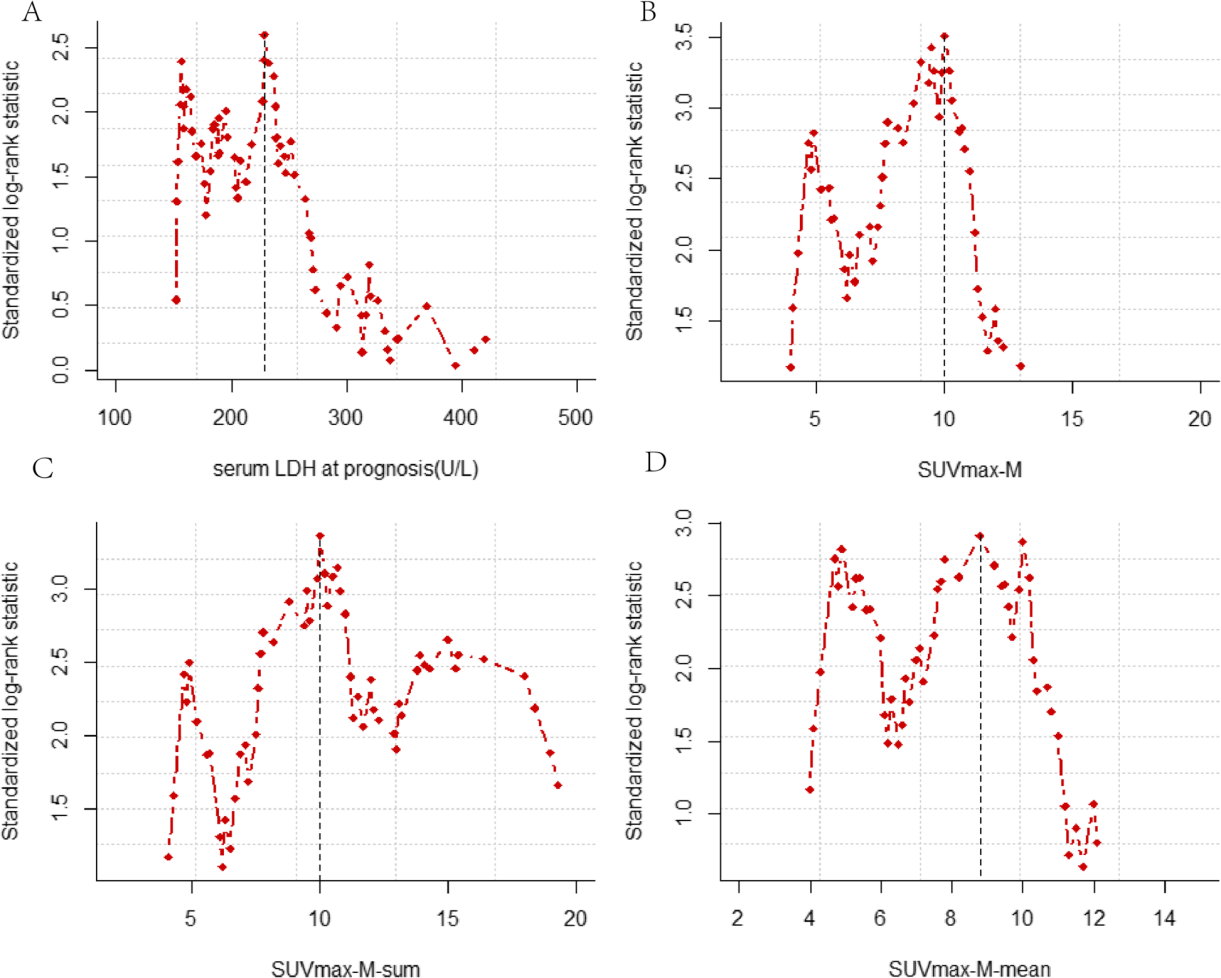
The optimal cutoff values for the variables of PET/CT and serum LDH at diagnosis

In the value of SUVmax-M (median: 9.25, range: 2–20.6), the association between survival hazard ratio (HR) and SUVmax-M was performed. Therefore, we chose the SUVmax-M = 10 as the optimal cutoff point due to the highest HR (Fig. 1). Accordingly, patients were classified into two cohorts: low-SUVmax-M (< 10), and high SUVmax-M (≥ 10). Similarly, the optimal values for the LDH at diagnosis (cutoff = 229), SUVmax-T (cutoff = 8), SUVmax-N (cutoff = 6.4), number of metastasis (cutoff = 4), SUVmax-M-sum (cutoff = 10), and SUVmax-M-mean (cutoff = 8.8) were determined and variables were converted as dichotomous variables (Fig. 2).

### Clinical characteristics and treatment outcomes in the high and low SUVmax-M group

**Fig. 3:**
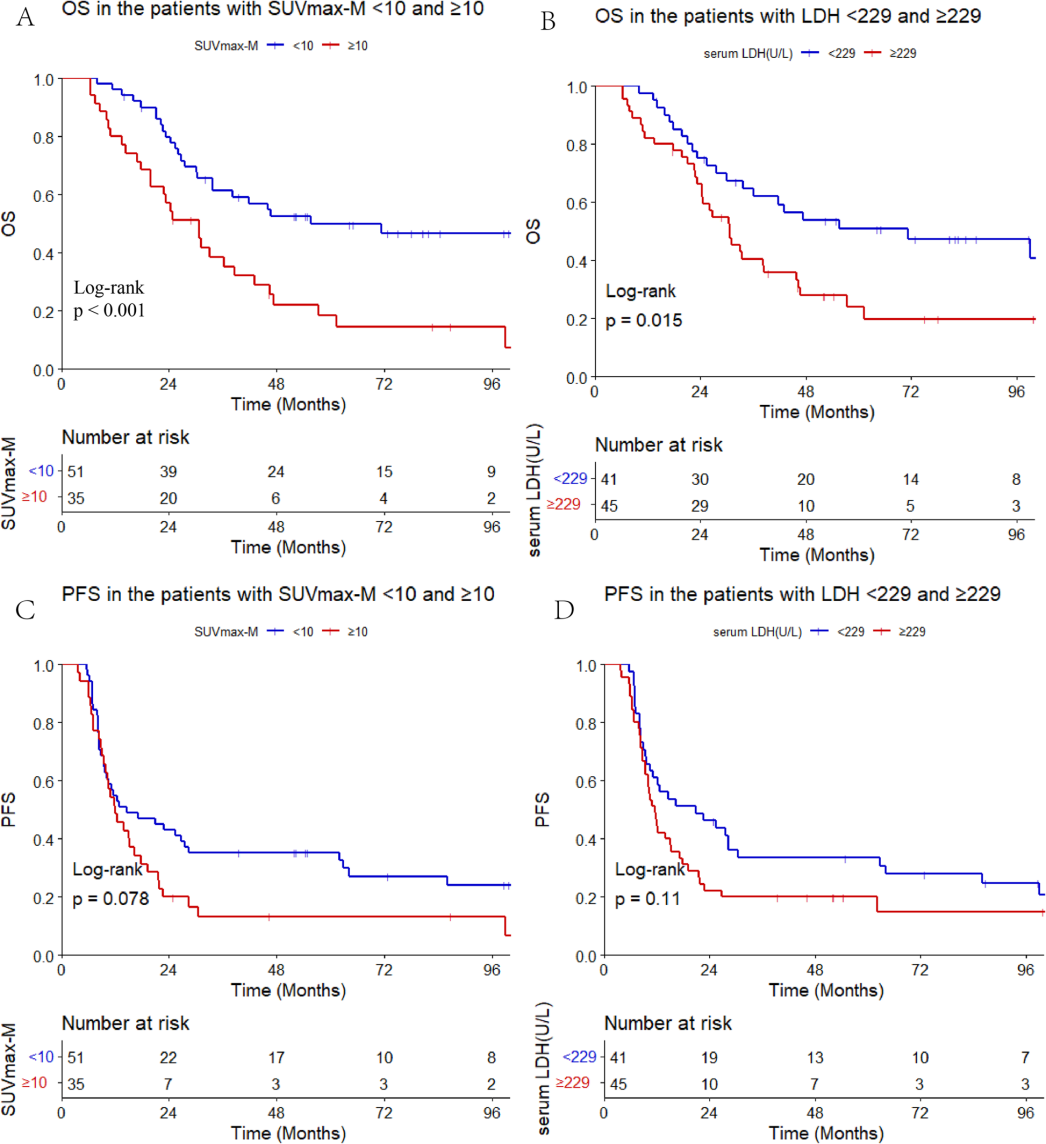
OS and PFS in the patients with SUVmax-M and serum LDH levels (**A**: OS in the SUVmax-M groups, **B**: OS in the LDH groups, **C**: PFS in the SUVmax-M groups, **D**: PFS in the LDH groups)

To illustrate the potential features associated with high SUVmax-M level, we compared the baseline characteristics between the SUVmax-M < 10 and ≥ 10 group. Higher SUVmax-M level was correlated with high SUVmax-N value (*P* < 0.001) and more metastatic sits (*P* = 0.068). More details about the features of the two groups are listed in Table 2. The 5-year OS in the SUVmax-M < 10 and ≥ 10 group was 49.8% and 18.4%, respectively (*P* < 0.001). Additionally, the 5-year OS in the low LDH (< 229) and high LDH (≥ 229) group was 50.9% and 23.9%, respectively (*P* = 0.015). However, the progression-free survival (PFS) was worse in the higher SUVmax-M and LDH groups but showed no statistical significance (*P* = 0.078, *P* = 0.11) (Fig. 3).

**Table 2:** Clinical and treatment characteristics in the SUVmax-M-low and SUVmax-M-high group

	SUVmax-M < 10	SUVmax-M ≥ 10	*P* value
	*N* = 51, %	*N* = 35, %	
Age	47.2 ± 11.4	44.3 ± 11.4	0.245
Gender			0.518
Male	7 (13.7%)	3 (8.57%)	
Female	44 (86.3%)	32 (91.4%)	
T stage			0.568
T1-2	22 (44.0%)	12 (35.3%)	
T3-4	28 (56.0%)	22 (64.7%)	
N stage			0.733
N0-1	7 (14.0%)	3 (8.82%)	
N2-3	43 (86.0%)	31 (91.2%)	
Metastatic sites	3.51 (2.71)	4.71 (3.12)	0.068
Presence of liver			1
No	38 (74.5%)	26 (74.3%)	
Yes	13 (25.5%)	9 (25.7%)	
Metastatic organs			0.437
Single	40 (78.4%)	24 (68.6%)	
Multiple	11 (21.6%)	11 (31.4%)	
SUVmax-T	10.8 ± 4.18	12.9 ± 5.56	0.061
SUVmax-N	9.76 ± 5.04	13.7 ± 5.57	0.001
Serum LDH	242 ± 99.8	469 ± 773	0.092
ACT			0.513
No	43 (84.3%)	32 (91.4%)	
Yes	8 (15.7%)	3 (8.57%)	
CCRT			0.577
No	43 (84.3%)	27 (77.1%)	
Yes	8 (15.7%)	8 (22.9%)	
IC circles	5.53 ± 0.97	5.51 ± 1.58	0.960

*Abbreviations: ACT* adjuvant chemotherapy, *CCRT* concurrent chemoradiotherapy, *IC* induction chemotherapy

### Selection of the variables in a predictive model

**Fig. 4:**
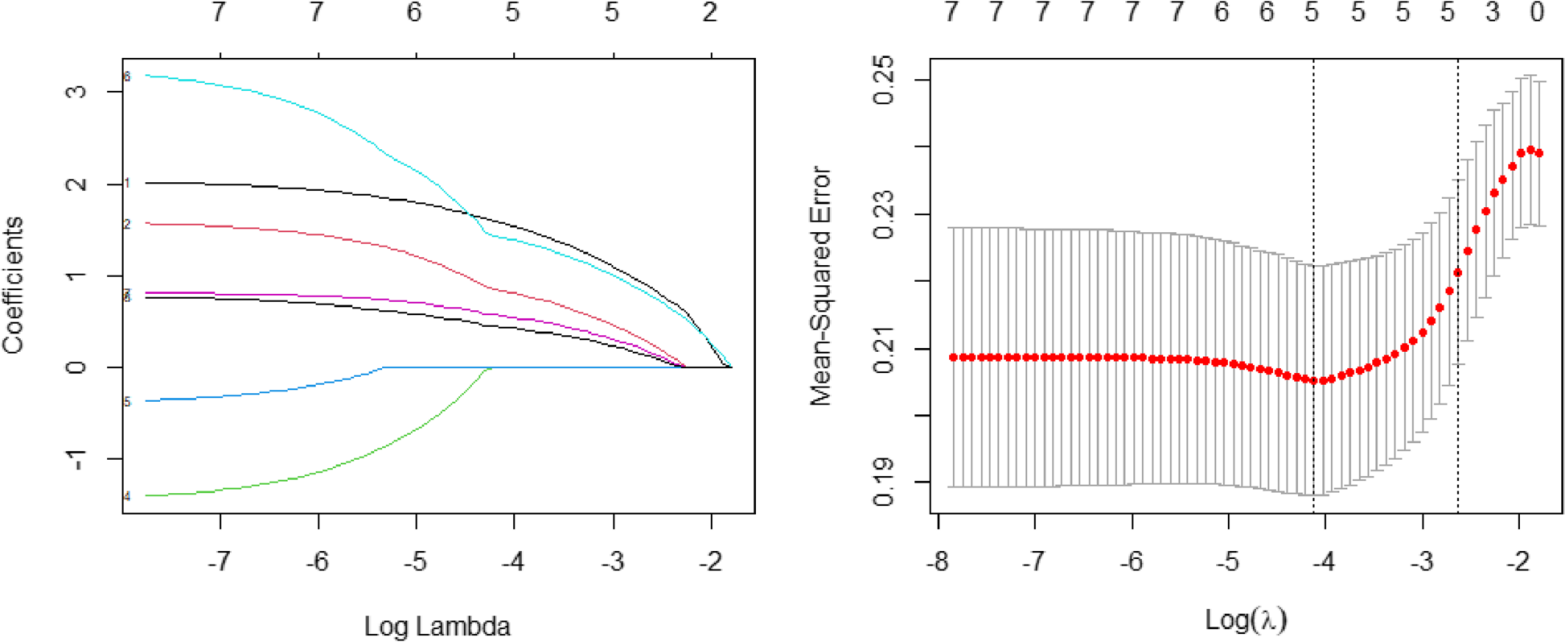
LASSO regression of the enrolling variables

In the univariable analysis, gender, number of metastasis, presence of liver, serum LDH, SUVmax-M, SUVmax-M-sum, and SUVmax-M-mean were found to be significantly associated with OS (Table 3). LASSO regression was conducted to remove nonsignificant variables via the regression coefficients penalizing the size of the parameters. After building the regression model using the seven variables, we chose the λ via the minimum criteria (Fig. 4). Consequently, five variables (gender, liver involvement, number of metastasis, LDH, and SUVmax-M) were selected and analyzed in the Cox regression model (Table 3).

**Table 3:** The univariate and multivariate analysis for OS in de novo mNPC

	Univariable analysis		Multivariate analysis	
	HR (95% CI)	*P* value	HR (95% CI)	*P* value
Age	1.01 (0.99–1.04)	0.395		
Gender		0.02		0.065
Female	Reference		Reference	
Male	5.36 (1.3–22.07)		3.81 (0.918–15.8)	
T stage		0.658		
T1-2	Reference			
T3-4	0.88 (0.51–1.53)			
N stage		0.072		
N1	Reference			
N2-3	2.92 (0.91–9.42)			
Liver involvement		0.03		0.039
No	Reference		Reference	
Yes	1.87 (1.06–3.31)		1.94 (1.031–3.64)	
Metastatic site		0.155		
Single	Reference			
Multiple	1.53 (0.85–2.75)			
Number of metastasis		0.016		
< 4	Reference			
≥ 4	1.94 (1.13–3.34)			
LDH		0.017		0.05
< 229	Reference		Reference	
≥ 229	1.96 (1.13–3.42)		1.81 (1.00–3.28)	
SUVmax-T		0.788		
< 8	Reference			
≥ 8	1.1 (0.56–2.13)			
SUVmax-N		0.126		
< 6.4	Reference			
≥ 6.4	1.71 (0.86–3.41)			
SUVmax-M		0.001		0.004
< 10	Reference		Reference	
≥ 10	2.49 (1.45–4.27)		2.10 (1.20–3.67)	
SUVmax-M-sum		0.002		
< 10	Reference			
≥ 10	2.39 (1.36–4.21)			
SUVmax-M-mean		0.009		
< 8.8	Reference			
≥ 8.8	2.07 (1.2–3.55)

### Identifications of significant predictors for OS in de novo mNPC

After the multivariate analysis with the variables from the results of LASSO regression, we found that liver involvement (*P* = 0.039, HR 1.94, 95% CI 1.031–3.64), higher serum LDH (≥ 229) (*P* = 0.05, HR 1.81, 95% CI 1.0–3.28), and higher SUVmax-M (≥ 10) (*P* = 0.004, HR 2.10, 95% CI 1.20–3.67) were independent prognostic predictors for poor OS (Table 3).

## Discussion

Patients presenting with synchronous metastasis at diagnosis can have diverse prognosis due to various metastatic burden and clinical features. In this study, we enrolled the patients who underwent initial ^18^F-FDG PET/CT to investigate the prognostic role of several features involving SUVmax levels in de novo mNPC. Interestingly, we found that higher level of SUVmax-M, liver involvement, and elevated serum LDH were independently associated with decreased OS in patients following chemotherapy plus LRRT.

Palliative chemotherapy was recommended as the first-line treatment approach for most disseminated malignancies. Several platinum doublet regimens have been employed in mNPC demonstrating favorable disease response rates ranging from 62.5% to 89.8% [19–21]. The phase III randomized study [22] has revealed the superior OS benefit of gemcitabine plus cisplatin (GP) and confirmed the GP as the first-line treatment for mNPC. Considering the high response to chemoradiotherapy, LRRT after effective chemotherapy was crucial for disease control. The results from public database have shown significant survival improvement of additional local radiotherapy to chemotherapy [23, 24]. Furthermore, the role of LRRT in de novo mNPC was illustrated by a phase III randomized trial [8]. Nonetheless, the optimal combination of chemoradiotherapy and patient selection is still inconclusive due to the heterogeneous outcome. Prognostication of the patients could be helpful in personalizing individual treatment strategy. Various clinical features and laboratory biomarkers were incorporated for building prognostic risk stratification of de novo mNPC [25–27]. However, the parameters of metabolism of metastatic lesions were not included.

Progression of distant metastatic lesions remained the major failure pattern for patients with de novo mNPC. Multiple metastatic organs were correlated with poor outcome [28] suggesting that higher metastatic burden increased the risk of disease progression. However, our results showed negative predictive impact of multiple involving organs in the univariable analysis (*P* = 0.115) may be due to the limited sample size. Furthermore, liver involvement was demonstrated as a significant predictor for worse OS in non-epidemic [28, 29] and epidemic area [26] which was consistent with our finding in the multivariate analysis (Table 3). Thus, liver involvement was integrated into the subdivision of stage M1 for de novo mNPC [30]. Moreover, the LDH was a key enzyme functioning in the anaerobic glycolysis suggesting the correlation of higher LDH level with tumor hypoxia, cancer progression and metastasis [31]. Elevated pretreatment serum LDH could predict poor survival in metastatic NPC treated by palliative chemotherapy [32, 33]. Additionally, higher level of LDH was correlated with worse OS in de novo mNPC [21] following chemotherapy with or without radiotherapy. Our study also illustrated the predictive role of high serum LDH (≥ 229 IU/L) in patients who received chemoradiotherapy.

The whole-body FDG PET/CT could provide functional data on tumor metabolism reporting higher sensitivity and accuracy than MRI in diagnosing initial NPC [34]. Interestingly, patients staged by FDG PET/CT showed better OS than those by MRI in treatment-naïve NPC [34]. Furthermore, the biological biomarker such as SUVmax represented glucose metabolic rate of tumor cells which indirectly indicates cancer burden. Advanced primary tumor and nodal stage were associated with worse OS in local NPC. Accordingly, previous studies have confirmed that higher SUVmax values of tumor and lymph nodes were correlated with poor survival in local NPC [12, 35]. Additionally, highest uptake value of the metastatic node could predict distant failure in locally advanced NPC [36]. Among the patients with synchronous metastasis at initial diagnosis, FDG uptakes could be measured in primary tumor, cervical lymph node and metastatic lesions. We defined the value of 10 as the cutoff point for SUVmax-M, and both the SUV-T and SUV-N were higher in the SUV-M ≥ 10 group, indicating the increased tumor aggression when detecting higher SUV-M level. A previous study evaluated the predictive value of SUVmax in de novo mNPC with or without locoregional radiotherapy in 2019 [37] but showed no independent prognostic value of SUVmax-M. However, the randomized prospective trial [8] in 2020 has verified the superior OS of locoregional radiotherapy plus chemotherapy for de novo mNPC. Additionally, the sum of SUVmax-M and average of SUVmax-M were also correlated with OS in metastatic tumor [16] which were not included in that study. Thus, our study evaluated all three variables (SUVmax-M-total, SUVmax-M-mead, SUVmax-M) and revealed the prognostic value in the univariable analysis. Nonetheless, SUVmax-M was the most significant variable chosen using the LASSO regression while SUVmax-M ≥ 10 was associated with poor survival in the multivariate analysis. Moreover, combining clinicopathological features together with imaging-derived information could strengthening the predictive value for helping clinical decision-making of patients with cancer [38]. In this study, we have demonstrated the potential role of integrating SUVmax-M and clinical information for stratifying prognostic risk of patients with de novo mNPC, and more effective biomarkers are to be explored.

Several limitations should be considered in this study. First, there is inevitably selection bias due to the retrospective design of the work. Second, our study enrolled the patients treated at one center and the sample size was still small which limited the effectiveness of the results. Lastly, some of the indicators like EBV-DNA levels, metabolic tumor volume (MTV), and total lesion glycolysis (TLG) were not included in our analysis. Due to the long-term period of eligible patients, the information of EBV-DNA was missing in over 50% of the patients. Additionally, MTV and TLG were parameters involving function and volume of tumors, which also showed potential prognostic value in NPC. Nonetheless, several retrospective studies have reported negative predictive role of MTV and TLG in NPC [39, 40]. In Contrast, SUVmax emerged with the advantages of accuracy, convenience and repeatability. Thus, prospective study is needed to verify the results and more effective biomarkers for de novo mNPC is to be explored.

## Conclusion

We performed this single-center analysis exploring the predictive value of SUVmax in metastatic lesions for de novo mNPC following chemotherapy with locoregional radiotherapy. Pre-treatment high level of serum LDH, SUVmax of metastatic lesion and liver involvement were independently associated with poor OS in the patients.

## Data Availability

Research data are not available at this time.
